# Astaxanthin Mitigates ADHD Symptoms in Spontaneously Hypertensive Rats via Dopaminergic Modulation and Brain–Gut Axis Regulation

**DOI:** 10.3390/molecules30071637

**Published:** 2025-04-07

**Authors:** Yueyang Leng, Ning Wu, Jing Wang, Lihua Geng, Yang Yue, Quanbin Zhang

**Affiliations:** 1 CAS and Shandong Province Key Laboratory of Experimental Marine Biology, Center for Ocean Mega-Science, Institute of Oceanology, Chinese Academy of Sciences, Qingdao 266071, China; lengyueyang20@mails.ucas.ac.cn (Y.L.); wuning@qdio.ac.cn (N.W.); jingwang@qdio.ac.cn (J.W.); lhgeng@qdio.ac.cn (L.G.); yueyang@qdio.ac.cn (Y.Y.); 2 Laboratory for Marine Biology and Biotechnology, Qingdao Marine Science and Technology Center, Qingdao 266071, China; 3 University of Chinese Academy of Sciences, Beijing 101408, China

**Keywords:** attention deficit hyperactivity disorder (ADHD), astaxanthin, dopamine (DA), atomoxetine, neuroprotection, gut microbiota

## Abstract

Attention Deficit Hyperactivity Disorder (ADHD) is a prevalent neurodevelopmental disorder that significantly impacts learning, daily functioning, and personal development. Astaxanthin (ASTA), a naturally occurring antioxidant, has garnered interest as a potential therapeutic agent for various diseases, particularly in mitigating oxidative stress. This study explores a novel application of ASTA in the context of ADHD, aiming to investigate its therapeutic effects and underlying mechanisms. Spontaneously hypertensive rats (SHRs), widely used ADHD model animals, were treated with ASTA (50/100 mg/kg/day) for three weeks, 5 mg/kg/day atomoxetine (ATO) as the positive, and Wistar Kyoto (WKY) rats as control. Behavioral improvements were assessed using the open field test (OFT) and the Morris water maze (MWM). Biochemical analyses were conducted to evaluate changes in the levels of various neurotrophic factors, while histological examinations were performed to assess neuroprotective effects. Additionally, the role of ASTA in the brain–gut axis was investigated. The behavioral symptoms of hyperactivity, anxiety, and impaired spatial memory in ADHD animals were mitigated by ASTA. This improvement is primarily attributed to the restoration of neurotransmitter levels, particularly dopamine (DA), achieved through the modulation of several critical components within the dopamine system, including dopamine receptor 1 (DR1), dopamine transporter (DAT), tyrosine hydroxylase (TH), and synaptic-associated protein 25 (SNAP-25). Additionally, regulating the serotonin transporter (SERT) and glial cell-derived neurotrophic factor (GDNF) supports the recovery of serotonin levels and facilitates optimal brain development. Furthermore, cerebellar cells were protected, and the structure of the intestinal microbiota was regulated. ASTA can mitigate ADHD symptoms in SHR through the modulation of the dopaminergic system, multiple neurotransmitters, neurotrophic factors, and the neuro-intestinal environment, which establishes ASTA as a promising nutraceutical candidate for adjunctive therapy in pediatric ADHD.

## 1. Introduction

Attention Deficit Hyperactivity Disorder (ADHD) is a neurodevelopmental disorder that typically emerges during childhood, affecting approximately 5.3% of school-aged children. It is generally identified at an early stage and is characterized by a constellation of core symptoms, including developmentally inappropriate levels of hyperactivity, impulsivity, and inattention, which can significantly impair daily functioning [1]. ADHD exhibits a high degree of heritability, with genetic contributions estimated to range from 70% to 80% [2]. Genome-wide association studies have identified several risk loci associated with ADHD; however, these loci account for only a portion of the disorder’s heritability [3]. Twelve significant loci linked to ADHD are involved in processes such as neurotransmission, neurogenesis, synaptogenesis, and receptor localization in synapses. Neurobiological factors include alterations in the dopaminergic and noradrenergic systems, which are essential for regulating attention and impulse control. Dysregulation of these neurotransmitter systems has been implicated in the pathophysiology of ADHD. The pathogenesis of ADHD is intricate and multifaceted, encompassing genetic predispositions, environmental factors, and neurobiological changes. Additionally, ADHD is often associated with neuroinflammation, oxidative stress, and gut microbiota disorders, although the causal relationships among these factors have yet to be conclusively determined [4,5,6].

Amphetamine (in 1937), Methylphenidate (in 1955), and atomoxetine (in 2002) have successively gained FDA approval for ADHD treatment. While effective, they carry substantial potential risks including weight loss, growth inhibition, and the development of tolerance and psychological dependence with long-term use [7,8,9]. Current dietary supplements for ADHD primarily include probiotics and neurotransmitter modulators, with limited efficacy [10,11]. The investigation of natural and efficacious food functional ingredients with neuroprotective and gut microbiome-improving effects may offer promising avenues for the development of treatment strategies for ADHD.

Astaxanthin (ASTA), an endogenously occurring carotenoid, has attracted significant scholarly attention due to its therapeutic potential in neurodegenerative diseases such as Alzheimer’s disease (AD), Parkinson’s disease (PD), Amyotrophic Lateral Sclerosis (ALS), and Huntington’s disease (HD) [12,13,14]. ASTA exhibits direct neuroprotective effects and modulates the body’s inflammatory state via the brain–gut axis, thereby restoring homeostasis. However, the current body of research is relatively limited regarding the efficacy of ASTA in treating neurodevelopmental disorders, particularly Attention Deficit Hyperactivity Disorder (ADHD). The etiology of ADHD is hypothesized to involve dysregulated levels of monoamine neurotransmitters, with a specific focus on dopaminergic deficiency. ADHD is characterized by structural and functional abnormalities in various brain regions, as well as gut microbiota dysbiosis.

Spontaneously hypertensive rats (SHRs) are widely used as an animal model for ADHD. They exhibit key characteristics of ADHD, including inattention, impulsivity, and hyperactivity, at the age of 4–10 weeks [15]. These symptoms gradually diminish over time, with SHRs developing stable hypertensive traits by 12 weeks of age. SHRs exhibit neurotransmitter level alterations at the physiological level similar to those observed in ADHD patients, as well as behavioral characteristics and a responsiveness to pharmacological treatments specific to ADHD, which further supports their validity as a model animal for the disorder [16]. In contrast, Wistar Kyoto (WKY) rats are commonly used as control animals. They provide critical insights into both behavioral and physiological aspects of ADHD [17]. The present investigation was designed to delineate the effects of ASTA on ADHD and to elucidate the underlying mechanisms of its action through a series of in vivo experiments utilizing young SHRs and WKY rats.

## 2. Results and Discussion

### 2.1. Alleviation of Hyperactivity and Anxiety Behavior by ASTA

**Figure 1:**
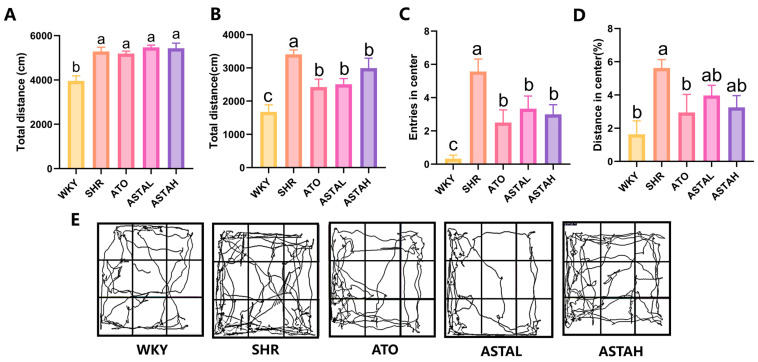
Hyperactivity behavior in the open field test. (**A**) The total distance traveled by animals in the open field before drug administration (F = 11.13). (**B**) The total distance traveled by animals after 14 days of drug administration (F = 10.03). (**C**) The number of animals entering the center (F = 8.47). (**D**) Animal movement distance (%) in the central area of the opening field (F = 3.61). (**E**) Representative pathways of animals in each group in the open field. All data are presented as mean ± SEM (*n* ≥ 6). The groups with the same letter are considered not significantly different, while groups with different letters are significantly different. Typically, lowercase letters indicate a significance level of *p* < 0.05. WKY, control group; SHR, untreated ADHD model group; ATO, positive group treated with 5 mg/kg/day atomoxetine; ASTAL, group treated with 50 mg/kg/day astaxanthin; ASTAH, group treated with 100 mg/kg/day astaxanthin.

Prior to ASTA administration, the activity status of the animals was assessed using the open field test (OFT), which demonstrated that SHRs exhibited significantly heightened hyperactivity and anxiety-related behaviors compared to WKY rats (Figure 1A). Consistent with the behavioral characteristics of ADHD and in agreement with previous studies, SHRs displayed increased exploratory activity in the open field compared to WKY rats [15]. No significant differences in activity levels were observed among the various SHR groups.

To assess the effects of ASTA on hyperactivity and anxiety in SHR, OFT was conducted after 14 days post-drug administration. Compared to SHR (untreated ADHD model group), WKY (the control group) exhibited a decreased inclination to explore novel and centrally open areas, leading to a reduction in their total distance in the open field (Figure 1B), the number entry into the center (Figure 1C), and the distance in center (Figure 1C), which aligns with the innate preference for dark corners and the cautious, timid nature typically observed in rodents [18].

In contrast, animals exhibiting ADHD-like symptoms demonstrate hyperactivity and anxiety in novel open environments, characterized by increased locomotor activity, particularly within the central area. Upon the administration of the drug, there was a notable decrease in the total distance traveled by these animals in the open field (Figure 1B), accompanied by a reduction in the frequency of entries into the central area (Figure 1C). Furthermore, the proportion of movement occurring within the central area was diminished (Figure 1D) significantly in ATO (the positive group treated with 5 mg/kg/day atomoxetine). Figure 1E presents the representative trajectories of animals from each group within the open field. These findings suggest that ASTA effectively mitigates hyperactive behavior in ADHD model animals in the open field. Additionally, at elevated concentrations, ASTA demonstrated an inhibitory effect on hyperactive and impulsive behaviors in the central area of SHR, which was comparable to that of ATO.

### 2.2. Effect of ASTA on Spatial Memory Ability

**Figure 2:**
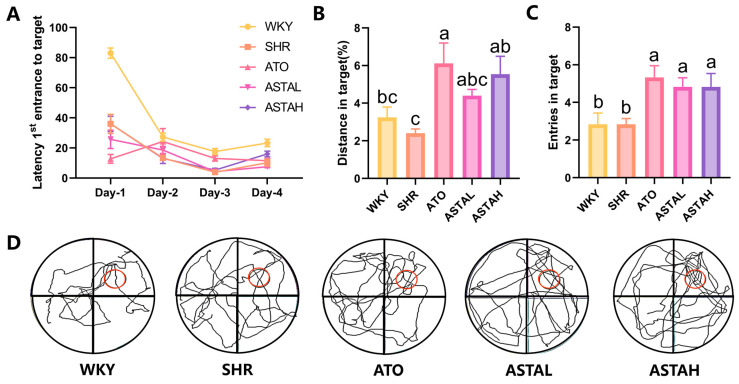
Spatial memory, learning ability, and attention in the Morris water maze test. (**A**) The time required for animals to enter the target in the position navigation test. (**B**) The number of times enters the target in the spatial navigation test (F = 4.76). (**C**) The movement distance in the target (F = 4.04). (**D**) Representative pathways of animals in spatial navigation. Red circular marker denotes the safe platform position. All data are presented as mean ± SEM (*n* ≥ 6). Groups with the same letter are considered not significantly different, while groups with different letters are significantly different. Typically, lowercase letters indicate a significance level of *p* < 0.05.

To assess the impact of ASTA on spatial memory and attention in SHR, the Morris water maze (MWM) test was conducted following the OFT. The MWM results are depicted in Figure 2. The animals were sequentially introduced into quadrants labeled I, II, III, and IV, each marked with distinct visual cues of different colors and shapes to facilitate spatial memory of the safe platform’s location. During the acquisition phase, the latency to locate the safe platform from different entry points was recorded. The results indicated a progressive reduction in latency with repeated trials, although it remained influenced by the distance between the entry point and the platform (Figure 2A). In the spatial probe test, the safe platform was removed, and SHR exhibited a lower frequency of active entries into the target zone and a reduced exploration ratio, indicating impaired spatial memory (Figure 2B). In contrast, animals in the ATO and ASTAH groups demonstrated a higher frequency of target zone entries and covered a greater distance within the zone, suggesting stronger retention of the safe platform’s location (Figure 2C). Representative swim paths are shown in Figure 2D, illustrating that the ATO, ASTAL, and ASTAH groups exhibited more trajectories concentrated in the third quadrant, where the safe platform was previously located (red circle). In contrast to the disorganized and erratic swimming patterns observed in the SHR group, the enhanced exploratory behavior in the target zone following drug administration suggests that these animals effectively utilized visual cues during the initial acquisition phase, facilitating rapid learning and retention of the safe platform’s location [19]. This observation indicates that both ATO and ASTA contribute to improvements in attention, learning capacity, and spatial memory in the ADHD model animals.

### 2.3. Regulation of Multiple Neurotransmitter Levels by ASTA

**Figure 3:**
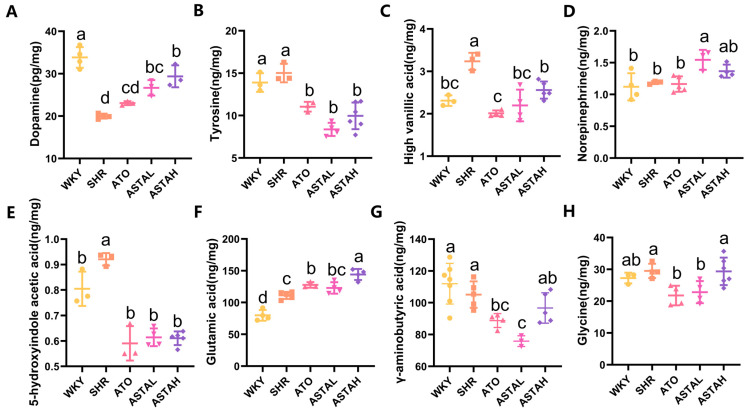
The regulation of neurotransmitter networks by astaxanthin. (**A**–**H**) Astaxanthin regulated dopamine (DA), tyrosine (Tyr), high vanillic acid (HVA), norepinephrine (NE), 5-hydroxyindole acetic acid (5-HIAA), glutamic acid (Glu), γ-aminobutyric acid (GABA), and glycine (Gly) in the PFC. This improved the dysregulation of the dopamine system, norepinephrine, serotonin system, and the balance of excitatory/inhibitory neurotransmitters. All data are presented as mean ± SEM (*n* ≥ 3). Groups with the same letter are considered not significantly different, while groups with different letters are significantly different. Typically, lowercase letters indicate a significance level of *p* < 0.05.

The neurochemical pathogenesis of ADHD manifests through monoaminergic dysregulation, particularly involving the hypofunction of dopaminergic/noradrenergic systems and serotonergic imbalance [6], compounded by disrupted excitatory/inhibitory neurotransmitter equilibrium. Liquid chromatography–mass spectrometry (LC-MS) analysis of prefrontal cortex (PFC) neurotransmitters, which is the central hub for cognitive control and attention regulation [5], revealed three critical findings. First, SHR animals exhibited a reduction in DA levels compared to WKY controls (Figure 3A), aligning with the known dopaminergic deficits in ADHD pathophysiology [20]. Notably, ATO and ASTA treatments dose-dependently restored DA concentrations. This recovery coincided with reduced tyrosine (Tyr) precursor levels and diminished homovanillic acid (HVA) metabolite (Figure 3B,C), suggesting enhanced Tyr-to-DA conversion through tyrosine hydroxylase (TH) activation and attenuated DA reuptake.

Additionally, neurotransmitter profiling uncovered ASTA’s multimodal neuromodulatory effects. While ASTAL increased norepinephrine (NE) levels (Figure 3D), both ATO and ASTA treatments decreased 5-hydroxyindoleacetic acid (5-HIAA) levels (Figure 3E), indicating enhanced serotonin retention through metabolic inhibition. Furthermore, glutamate (Glu) levels increased in treated groups (Figure 3F), reversing the cortical excitatory deficit implicated in ADHD’s attention deficits [21,22,23]. Conversely, the inhibitory neurotransmitters GABA and glycine decreased (Figure 3G,H), rebalancing the cortical E/I ratio critical for cognitive processing.

These findings collectively demonstrate that pharmacological intervention rectifies ADHD-associated neurochemical imbalances through three convergent mechanisms, including augmenting monoamine synthesis via precursor utilization (Tyr/DA) while inhibiting catabolic pathways (DA/HVA), modulating NE and serotonin recycling and restoring excitatory/inhibitory equilibrium (Glu/GABA) [24]. The dose-dependent normalization of dopaminergic signaling, coupled with glutamatergic enhancement and serotonergic stabilization, provides mechanistic rationale for ASTA’s behavioral efficacy observed in OFT and MWM assays.

### 2.4. Regulation of the Dopamine System by ASTA

**Figure 4:**
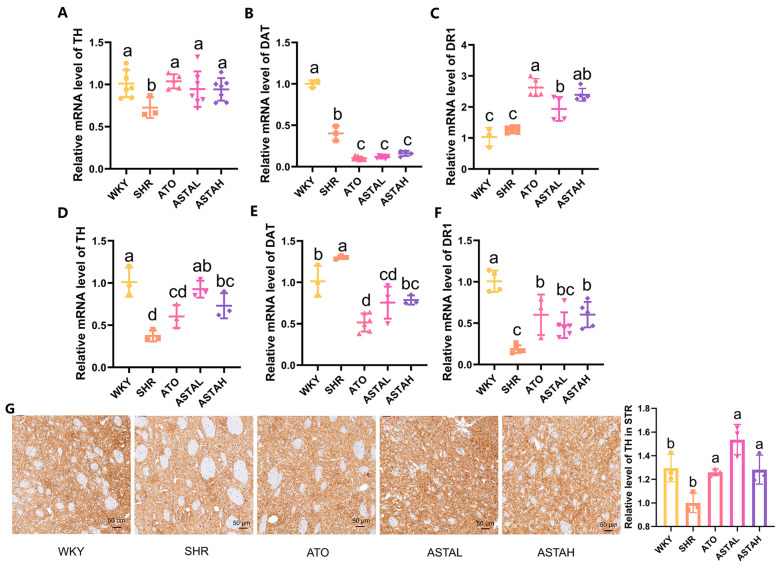
ASTA’s regulation of the dopamine system. (**A**–**C**) ASTA regulated tyrosine hydroxylase (TH), dopamine transport (DAT), and dopamine receptor 1 (DR1) levels in the PFC. (**D**–**F**) ASTA regulated transcription levels of TH, DAT, and DR1 in the STR. (**G**) ASTA regulated the expression level of TH in the STR (Scale bar = 50 μm). All data are presented as mean ± SEM (*n* ≥ 3). Groups with the same letter are considered not significantly different, while groups with different letters are significantly different. Typically, lowercase letters indicate a significance level of *p* < 0.05.

The centrality of the dopaminergic pathway in the pathophysiology of ADHD is underscored by ASTA’s multimodal regulation of DA synthesis, reuptake, and signaling [25]. Quantitative analysis revealed neuromodulation, including the upregulation of TH in the PFC and striatum (STR) (Figure 4A,D), corroborated by immunohistochemical verification of enhanced TH protein levels in STR dopaminergic terminals (Figure 4G). This transcriptional activation of the rate-limiting DA synthesis enzyme indicates restored DA production capacity. Additionally, DA reuptake dynamics were modulated through dopamine transporter (DAT) downregulation, with treated groups showing reduced DAT mRNA in the PFC and STR (Figure 4B,E). The attenuated presynaptic recapture mechanism, in synergy with TH upregulation, enhances synaptic DA availability [26]. Furthermore, dopamine receptor 1 (DR1) expression increased in both regions (Figure 4C,F), suggesting enhanced post-synaptic signal transduction [25]. This triad of effects augmented synthesis, prolonged synaptic presence, and strengthened receptor activation, establishing a self-reinforcing mechanism for dopaminergic signaling restoration.

Notably, the primary source of DA in the brain are the dopaminergic neurons located in the substantia nigra and ventral tegmental area [27]. These neurons project their axons primarily to the STR, where they are subsequently transported to the prefrontal cortex [28]. The coordinated regulation across the PFC-STR implies system neuromodulation. The TH protein, increased in the STR, particularly highlights ASTA’s capacity to rescue nigrostriatal pathway dysfunction. These molecular changes translate into improved cognitive enhancement in learning and memory tasks.

### 2.5. Regulation of Neural Pathway Factor Transcription Levels by ASTA 

**Figure 5:**
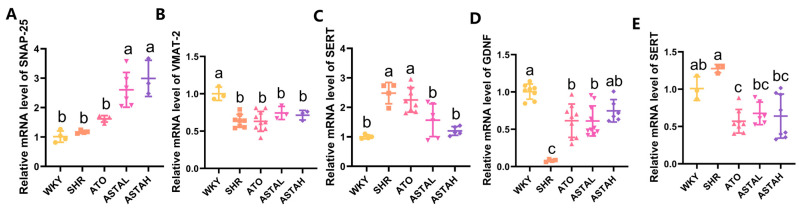
Astaxanthin regulated transcription levels of key factors of neural pathways in the PFC and STR. (**A**–**D**) Astaxanthin regulated synaptic-associated protein 25 (SNAP-25), vesicular monoamine transporters-2 (VMAT-2), serotonin transporter (SERT), and glial-cell-line-derived neurotrophic factor (GDNF) in the PFC. (**E**) Astaxanthin regulated SERT in the STR. All data are presented as mean ± SEM (*n* ≥ 3). Groups with the same letter are considered not significantly different, while groups with different letters are significantly different. Typically, lowercase letters indicate a significance level of *p* < 0.05.

The synaptic release machinery analysis revealed ASTA’s selective modulation of dopaminergic transmission components [25]. Quantitative profiling demonstrated significant dose-dependent upregulation of SNAP-25 (Figure 5A), the synaptic vesicle fusion protein critical for DA exocytosis, while vesicular monoamine transporter (VMAT-2) expression remained unchanged across treatment groups (Figure 5B). This differential regulation indicates ASTA specifically enhances dopamine vesicular release without affecting general monoamine storage mechanisms.

A concurrent downregulation of serotonin transporter (SERT) mRNA levels was observed in both the PFC (Figure 5C) and STR (Figure 5E), consistent with the reduced 5-HIAA metabolite levels detected in LC-MS analyses (Figure 3E). These coordinated changes suggest a dual mechanism for monoamine modulation: increased synaptic serotonin retention through SERT inhibition, coupled with potential release enhancement via SNAP-25 upregulation [29].

Notably, glial cell line-derived neurotrophic factor (GDNF) expression showed marked elevation in drug-treated groups (Figure 5D). This neurotrophic effect, particularly pronounced in ASTA groups compared to ATO groups, implies synergistic support for dopaminergic neuron survival and functional maintenance.

### 2.6. Histopathological Alterations in the Cerebellum Induced by ASTA

**Figure 6:**
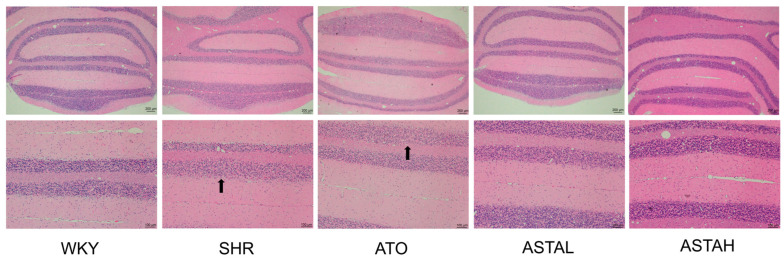
Histopathology of Purkinje cell layer in cerebellum. Scale bar = 200 μm (up), 100 μm (below). (→: Purkinje cells are sparsely arranged and have irregular boundaries).

The neuroprotective potential of ASTA extends to cerebellar preservation, particularly relevant given the established correlation between cerebellar morphological abnormalities and ADHD-related motor deficits [18]. Histopathological evaluation via H&E staining revealed that SHR group exhibited cerebellar granular layer hypoplasia with disorganized Purkinje cell architecture and nucleolar blurring (Figure 6), morphological hallmarks consistent with ADHD-associated cerebellar dysfunction. While ATO treatment induced pallor in cellular staining suggestive of subcellular stress response, ASTA administration restored granular layer cellularity with sharply defined nucleoli and intact Purkinje cell dendrites. This structural preservation aligns with ASTA’s documented antioxidant capacity in neurodegenerative models, here specifically mitigating cerebellar oxidative damage. The observed cytoarchitectural rescue likely underlies functional improvements in motor coordination and spatial memory—Purkinje cell-dependent processes frequently impaired in ADHD. Critically, the differential response between ASTA and ATO highlights compound-specific neuroprotective profiles, where ASTA’s pleiotropic antioxidant actions complement its neuromodulatory effects.

### 2.7. Regulation of Intestinal Microbiota by ASTA

**Figure 7:**
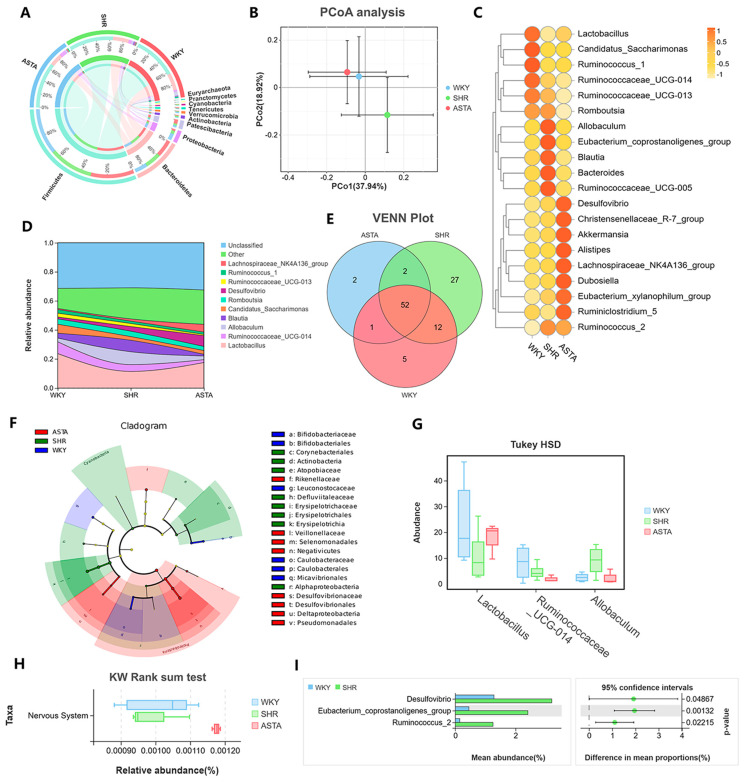
Structure and abundance of gut microbiota. (**A**) Circos species diagram (phylum level). (**B**) β diversity analysis (genus level). (**C**) Species abundance heat map (genus level). (**D**) Species distribution river map (genus level). (**E**) VENN Plot (family level). (**F**) LEfSe analysis (family level). (**G**) Tukey HSD rank sum test for indicator species (genus level). (**H**) KW rank sum test for functional analysis. (**I**) Welch’s *t*-test for indicator species (family level).

An imbalance in the proportion of Firmicutes (F) and Bacteroidetes (B) indicates a disruption in the equilibrium of the microbiota, potentially leading to significant pathological alterations [30]. Figure 7A illustrates the species composition at the phylum level. Firmicutes bacteria constituted the largest proportion, comprising 63.02% in the WKY group, 64.69% in the SHR group, and 67.12% in the ASTAL group. Following Firmicutes, Bacteroidetes accounted for 24.82% in the WKY group, 23.21% in the SHR group, and 18.06% in the ASTAL group. The F/B ratio was higher in the ASTA group (3.72 ± 0.19) compared to the SHR group (2.78 ± 0.21), indicating a reduced likelihood of inflammatory bowel disease [31].

The Circos diagram of species at the phylum level reflects the abundance of each phylum in the groups (Figure 7A). Firmicutes had the highest level in the ASTA group. The results of the PCoA analysis indicated a significant difference in the gut microbiota between the SHR group and the ASTA group, suggesting that ASTA has a regulatory effect on the gut microbiota (Figure 7B). At the genus level, ASTA upregulated *Akkermansia*, *Alistipes*, *Dubosiella*, and *Eubacterium* (Figure 7C). *Alistipes* and *Akkermansia* have been shown to have clinical effects on liver fibrosis, cancer immunotherapy, cardiovascular disease, and neurobiological effects [22]. Additionally, *Duosiella* and *Eubacterium*, the high-yield butyric acid producer, positively affect long-term memory and cognitive impairment [32]. ASTA downregulated the *Ruminococcus* which is an inflammatory marker bacterium, compared to the SHR group.

The distribution river map at the genus level reveals that the WKY and ASTA groups had more *Lactobacillus* and less *Blautia* compared to the SHR group. This suggests that ASTA regulated the intestinal environment to achieve lower levels of inflammation (Figure 7D). The VENN plot indicates that there were 52 shared microbial communities among the groups at the family level (Figure 7E). Within the SHR group, there were 27 distinct microbial communities, but only 2 shared with the ASTA group, indicating a significant difference following ASTA treatment. The lower levels of *Blautia* and *Lachnospiraceae_NK4A136_group* suggest a reduced potential risk of Crohn’s disease and ulcerative colitis. Lefse analysis yielded biomarkers for each group (Figure 7F). In the SHR group, Corynebacteriales, Atopobiaceae, and Alphaproteobacteria had higher levels. These are human opportunistic pathogens that may cause immune dysfunction and local infections. Rikenellaceae and Veillonellaceae, which regulate immune function, were relatively high in the ASTA group. Rikenellaceae is one of the strains that distinguish schizophrenia patients from the healthy population. Veillonellaceae may play a protective and supportive role in early childhood immune system development.

The KW rank sum test showed that ASTA downregulated *Weissella* and *Fusicatenibacter*. *Weissella* is a potentially beneficial probiotic and it is highly relative in cryptococcal meningitis [33]. The Tukey HSD test results indicated that ASTA significantly upregulated *Lactobacillus* and downregulated *Ruminococcaceae_UGG-014* and *Allobaculum* (Figure 7G). *Ruminococcaceae* and *Allobaculum* are the greatest differences between the control group and depressed mice, suggesting that they may be biomarkers for neurodevelopmental diseases. Tax4Fun functional analysis revealed that ASTA treatment significantly upregulated the microorganisms that affect the nervous system (Figure 7H). Meanwhile, there were lower levels of *Desulfovibrio*, a sulfate-reducing bacterium, and *Ruminococcus_2* compared to the SHR group (Figure 7I). This suggested that WKY rats may be a potential model for ASD or depression disorder.

Research indicates that enhancements in the gut microbiota can impact brain function and behavior by modulating dopamine synthesis and metabolism [34], as well as supporting the survival of dopaminergic neurons [35]. ASTA, recognized for its potent antioxidant properties, exerts its effects through anti-inflammatory and antioxidative pathways and by regulating gut microbiota, thereby influencing metabolic and immune functions [36]. In comparison to dietary polyphenols [11], ASTA demonstrates greater efficacy in modulating the abundance of gut microbiota, such as Bifidobacterium, which is linked to the production of short-chain fatty acids. This regulation enhances immune function, mitigates neuroinflammation, and influences neurotransmitter secretion and transmission, ultimately contributing to the homeostatic balance of neurotransmitters in the brain.

## 3. Materials and Methods

### 3.1. Reagents

Astaxanthin extracted from *Haematococcus pluvialis* was provided by Yunan Alphy Biotech Co., Ltd., China.

Atomoxetine hydrochloride was purchased from LILLY (LILLY DEL CARIBE Inc., Puerto Rico, USA); Standards of neurotransmitters, including dopamine (DA), tyrosine (Tyr), homovanillic acid (HVA), norepinephrine (NE), 5-hydroxyindoleacetic acid (5-HIAA), acetylcholine (ACh), glutamate (Glu), γ-aminobutyric acid (GABA), and glycine (Gly), were purchased from Shanghai Yuanye (Shanghai Yuanye Bio-Technology Co., Ltd., Shanghai, China). The immunohistochemistry antibodies used were TH (sc-25269) antibodies from Santa (Santa Cruz Biotechnology, CA, USA).

### 3.2. Animals and Drug Administration

Ten male Wistar Kyoto (WKY) wild-type rats and forty male spontaneously hypertensive rats (SHRs), aged 4 weeks, were sourced from Beijing Vital River Laboratory Animal Technology Co., Ltd. (Beijing, China, Permit No. SCXK(Jing)2021-0006; experimental unit usage license number: SYXK(Lu) 2020 0018). The animals were maintained in a controlled environment, with stringent parameters for temperature (22 ± 3 °C), relative humidity (60 ± 5%), and a regulated light–dark cycle (12:12 h). The animal experiment protocols were conducted following the ‘Guide for the Care and Use of Laboratory Animals’ (8th edition, National Research Council of the National Academies, 2011) and were approved by the Qingdao University Laboratory Animal Welfare Ethics Committee (Ethics Approval Number: 20211126SHR10020220115006) on 21 October 2021.

Following a 5-day acclimatization period to the experimental conditions, the WKY rats were assigned to the control group (WKY) and received a daily gavage of saline solution at a volume of 1 mL per 100 g of body weight. The SHRs were randomly allocated into four distinct treatment groups, each consisting of ten animals. The model group (SHR) received a comparable volume of saline via gavage. The positive control group (ATO) was administered with atomoxetine at a dosage of 5 mg/kg/day via gavage. The ASTAL and ASTAH groups were given astaxanthin (ASTA) at dosages of 50 mg/kg/day and 100 mg/kg/day, respectively, via gavage. The concentration of ATO was determined based on the recommended dose of the atomoxetine hydrochloride product, adjusted according to the drug absorption conversion factor between rats and humans. The concentration of ASTA was optimized concerning its application in other neurological disorders [37,38] and our previous experiment data. The daily morning dosing regimen was sustained for 3 weeks, with an open field test conducted after the second week. The Morris water maze test was performed in the subsequent week.

In the subsequent tissue preparation phase, designed to evaluate the neurobiological effects, the animals were anesthetized with pentobarbital (100 mg/kg i.p.) followed by transcardial perfusion. Brain tissues were extracted from four animals per group and postfixed by immersion in a 4% paraformaldehyde (PFA) solution for subsequent immunohistochemical analysis. Additionally, tissues from six animals per group were procured for the dissection of the prefrontal cortex (PFC) and striatum (STR). Concurrently, fecal samples were collected from each animal in sterile EP tubes for subsequent analysis of microbial composition.

### 3.3. Behavioral Tests

The open field test (OFT) was administered both prior to the commencement of pharmacological intervention and following a 14-day treatment period. Subsequent to the latter OFT, the Morris water maze (MWM) was conducted.

The OFT, a standardized ethological procedure, assesses the autonomous behavior, exploratory tendencies, and anxiety levels of animals in a novel environment [39]. The OFT apparatus measured 50 × 50 × 45 cm and was subdivided into nine equal squares. At the onset of the assay, rats were positioned at the center of the chamber, and their movements were recorded for a duration of 5 min using a video camera. The total distance traveled (cm), number of entries into the central area (*n*), and the percentage of distance traveled within the central area (%) were then analyzed using the Smart V3.0.0.6 software (Reward Life Science Co., Ltd., Shenzhen, China).

The MWM test is a widely employed technique for assessing the cognitive and memory capabilities of animals [19]. The apparatus comprises a circular pool with a radius of 90 cm and a height of 50 cm, filled with black water maintained at a temperature of 25 °C. It includes a square platform measuring 10 cm in radius and 40 cm in height, as well as an image collection system. The water maze is segmented into four quadrants, with the platform situated in the third quadrant. On the initial day of the MWM test, the platform is positioned above the water surface. Rats are introduced into each quadrant, oriented towards the pool wall, and allowed 2 min for exploration. During the position navigation phase, the platform is submerged 2 cm below the water surface. In the subsequent Spatial Exploration phase, the platform is removed, permitting the animals to explore for 60 s. The latency to locate the platform (s), the exploration distance within the target platform area (cm), and the number of entries into the target area (*n*) are recorded to assess the animals’ spatial memory performance.

### 3.4. Liquid Chromatography–Mass Spectrometry (LC/MS) Analysis

Neurotransmitters were isolated from the dissected prefrontal cortex (PFC) tissue through homogenization with a tenfold volume of a methanol and acetonitrile mixture (volume ratio 1:1) [40]. The homogenate was subsequently subjected to centrifugation at 12,000 rpm for 10 min under precooled conditions. To ensure analytical precision, 200 nM of 2-pyridyl acetic acid hydrochloride was incorporated as an internal standard (IS). The liquid chromatography–mass spectrometry (LC/MS) system consists of two components. The high-performance liquid chromatograph (30AD, Shimadzu Corporation, Kyoto, Japan) was equipped with an ACQUITY UPLC BEH Shield RP18 column (2.1 × 10 mm, 1.7 μm) maintained at 25 °C. Mobile phase A comprised a 0.1% aqueous formic acid solution, while mobile phase B was acetonitrile. The optimized gradient elution protocol was as follows: 0–2 min, 2% B; 2–3 min, 2% B to 90% B; 3–5 min, 90% B; 5–6 min, 90% B to 2% B; 6–10 min, 2% B. A volume of 10 microliters of the supernatant was injected, facilitating the separation of neurotransmitters. The brain samples were ionized into fragments and analyzed using a Triple Quad 4500 mass spectrometer (SCIEX Framingham, Massachusetts, USA) operating in positive ion mode with electrospray ionization (ESI). Critical parameters were optimized through an automated run, as detailed in Table 1. All data were processed using Analyst 1.6.2 software (AB SCIEX, USA).

### 3.5. Quantitative Real-Time PCR

Total RNA was extracted from the PFC and STR by a FastPure^®^ Cell/Tissue Total RNA Isolation Kit V2 (Vazyme Biotech Co., Ltd., Nanjing, China). cDNA was synthesized from 300 ng of total RNA using HiScript^®^ III RT SuperMix for qPCR (+gDNA wiper) from Vazyme Biotech Co., Ltd. The sequences of the PCR primers are listed in Table 2. qRT-PCR was performed on a QuantStudio 6 Flex Real-Time PCR System (Applied Biosystems, Waltham, MA, USA) using ChamQ Universal SYBR qPCR Master Mix (Vazyme Biotech Co., Ltd.). Relative gene expression levels of tyrosine hydroxylase (TH), dopamine transporter (DAT), dopamine receptor 1 (DR1), synaptic-associated protein 25 (SNAP-25), serotonin transporter (SERT), glial-cell-line-derived neurotrophic factor (GDNF), and vesicular monoamine transporter-2 (VMAT-2) were determined using the 2^−ΔΔCt^ method. GAPDH was utilized as the internal control and reference gene [41].

### 3.6. Histological Staining and Immunohistochemical Staining

To assess the histological changes and the expression of TH in the STR, the brain tissue were dehydrated, embedded in paraffin, and cut into 5 μm thick sections after fixation in 4% PFA for 48 h at 4 °C [42]. The sections were stained with hematoxylin and eosin (H&E). For immunohistochemical staining, brain tissues slides were treated to repair the antigens by incubating them with 3% hydrogen peroxide for 10 min and then blocking them with 1% BSA for 1 h at room temperature. The slides were incubated with TH (diluted at 1:1000) overnight at 4 °C. After being washed with PBS three times, the sections were incubated with rabbit secondary antibodies for 2 h at 25 °C and then counterstained with DAB staining. These stained slides were digitized using a digital slide scanner, and the image analysis was conducted in ImageJ 1.54f (National Institutes of Health, Bethesda, MD, USA).

### 3.7. Analysis of Gut Microbiota

For gut microbiota detection using fecal samples from the colons of the animals, the experimental steps were as follows: DNA was extracted from feces, and the 16S rDNA target region of the ribosomal RNA gene was amplified by polymerase chain reaction. Primers 341 F (5′-CCTAYGGGRBGCASCAG-3′) and 806 R (5′-GACTACNNGGGTATCTAAT-3′) were used to amplify the bacterial 16S rDNA (341 to 806) of the V3 + V4 region. Amplicons were purified and paired-end sequenced on an Illumina platform (PE250), and the raw reads were deposited into the NCBI Sequence Read Archive database. Bioinformatics analysis was performed using a real-time interactive online data analysis platform (http://www.omicsmart.com, accessed on 6 March 2024 ) [11].

### 3.8. Statistical Analysis

The results were presented as mean ± SEM for at least three replicates using GraphPad Prism 8 (GraphPad Software, San Diego, CA, USA). Parametric data were analyzed using IBM SPSS software version 25.0 (IBM SPSS, Armonk, New York, USA). Normality was tested by Shapiro–Wilk test. For datasets with a normal distribution, one-way ANOVA followed by Student–Newman–Keuls post hoc tests was used to compare significant differences between groups. All results were considered statistically significant at *p* < 0.05.

## 4. Conclusions

This study confirms the efficacy of ASTA in alleviating ADHD-like phenotypes. Its anti-ADHD effects emerge through a coordinated regulation of dopaminergic circuit plasticity, neuroprotection, and gut microbiota–neuroinflammation crosstalk. Such multi-system synergy establishes ASTA as a promising nutraceutical candidate for adjunctive therapy in pediatric ADHD, particularly given its dual action on central neurotransmission and homeostatic restoration.

## Figures and Tables

**Table 1:** List of multiple LC/MS reaction monitoring parameters.

Analyte	Q1 Mass (*m/z*)	Q3 Mass (*m/z*)	DP (V)	CE (V)
DA	154.1	137.2	50	15
NE	170.1	152.2	90	10
5-HIAA	192.1	146.1	60	20
HVA	183.1	137.1	90	25
ACh	146.1	87.1	80	20
GABA	104	87.1	30	15
Tyr	182	123	50	40
Glu	148.1	84.1	80	15
Gly	76	30	40	20
IS	137.9	120.1	25	25

As listed, precursor and product ions (Q1, Q3) with specificity for the target molecule were selected for quantitation and identification. The critical parameters (DP, CE) were optimized by automated run for identifying ions in the LC/MS system.

**Table 2:** DNA sequences of primers used in qRT-PCR.

	Forward Primer	Reverse Primer
GAPDH	TTCACCACCATGGAGAAGGC	CTCGTGGTTCACACCCATCA
TH	TCCCAGGACATTGGACTTGC	AAGCCTTCAGCTCCCCATTC
DAT	GTCACCAACGGTGGCATCTA	AATTGCTGGACGCCGTAGAA
DR1	CACCTGAGGTCCAAGGTGAC	AAGGACCCAAAGGGCCAAAA
SNAP-25	AGTCACACAAGGCTACCAGC	GTGGCTTTGGAGGCAAACAG
SERT	CCGTCATCTGCATCCCTACC	ATGTCCCCACACGGGATTTC
GDNF	CGCTGACCAGTGACTCCAAT	CGCCGCTTGTTTATCTGGTG
VMAT-2	CCCTGCACCTCCTCCAAATC	GATAGCTTCTGGTCCCGCTC

## Data Availability

The datasets generated and/or analyzed during the current study are available from the corresponding author on reasonable request.

## Abbreviations

The following abbreviations are used in this manuscript:

ADHD	Attention Deficit Hyperactivity Disorder
ASTA	astaxanthin
OFT	open field test
MWM	Morris water maze
DA	dopamine
DR1	dopamine receptor 1
DAT	dopamine transporter
TH	tyrosine hydroxylase
SNAP-25	synaptic-associated protein 25
SERT	serotonin transporter
GDNF	glial-cell-line-derived neurotrophic factor
5-CSRTT	5-choice serial reaction time test
DSM-5	Diagnostic and Statistical Manual of Mental Disorders
AD	Alzheimer’s disease
PD	Parkinson’s disease
ALS	Amyotrophic Lateral Sclerosis
HD	Huntington’s disease
SHR	spontaneously hypertensive rat
WKY	Wistar Kyoto
Tyr	tyrosine
HVA	homovanillic acid
NE	norepinephrine
5-HIAA	5-hydroxyindoleacetic acid
Ach	acetylcholine
Glu	glutamate
GABA	γ-aminobutyric acid
Gly	glycine
PFA	paraformaldehyde
PFC	prefrontal cortex
STR	striatum
LC/MS	liquid chromatography–mass spectrometry
IS	internal standard
ESI	electrospray ionization
VMAT-2	vesicular monoamine transporter-2
H&E	hematoxylin and eosin
MAO-B	Monoamine Oxidase B
LTP	long-term potentiation
qRT-PCR	quantitative real-time polymerase chain reaction
IHC	immunohistochemistry
CE	cerebellum
DOAJ	Directory of Open Access Journals
TLA	three-letter acronym
LD	linear dichroism
